# Efdamrofusp alfa: an insight into the novel drug and its use in age-related macular degeneration

**DOI:** 10.1186/s40942-025-00685-2

**Published:** 2025-09-29

**Authors:** Shree Rath, Arwa Amer Ibrahim, Arashdeep Singh, Arghadip Das, Sayed Mansoor Sediqi, Najia Ali Khan, Krisha Panchal, Safwan Masaud Mian

**Affiliations:** 1 All India Institute of Medical Sciences, Bhubaneswar, India; 2 University of Sharjah, Sharjah, United Arab Emirates; 3 Government Medical College, Amritsar, Punjab India; 4 Nilratan Sircar Medical College and Hospital, Kolkata, West Bengal India; 5 American University of Afghanistan, Kabul, Afghanistan; 6 Khyber Medical College, Peshawar, Pakistan; 7 Smt. NHL Municipal Medical College, Ahmedabad, Gujarat India

**Keywords:** Efdamrofusp alfa, Bispecific protein, Age-related macular degeneration (AMD), Immunotherapy, Clinical trials

## Abstract

**Purpose:**

Age-related macular degeneration (AMD) is a leading cause of irreversible blindness in older adults, with its prevalence rising globally. This review aims to explore the potential of Efdamrofusp alfa (EA), a novel bispecific decoy receptor fusion protein targeting both VEGF and complement pathways, in treating neovascular AMD (nAMD).

**Methods:**

A comprehensive literature search was conducted across PubMed, Cochrane and Embase till March 2025 to find articles evaluating the efficacy of EA in the treatment of neovascular AMD. Observations from early pre-clinical studies and clinical trials were analyzed to determine the efficacy and safety of EA.

**Results:**

A total of five preclinical and clinical studies were included, encompassing 66 animal subjects and 880 human participants. Efdamrofusp alfa (IBI302) neutralizes both C3b/C4b and VEGF, demonstrating anti-angiogenic effects in preclinical models. Clinical trials examined intravitreal doses ranging from 0.05 mg to 4.00 mg. EA showed efficacy in reducing central retinal thickness and improving visual acuity, with a safety profile comparable to existing anti-VEGF treatments. Treatment-emergent adverse events (TEAEs) included conjunctival hemorrhage, ocular hypertension, and keratitis, which were similar to those observed with other intravitreal anti-VEGF drugs. The drug demonstrated noninferiority to aflibercept in improving best-corrected visual acuity (BCVA) and significantly reduced central subfield thickness.

**Conclusions:**

Efdamrofusp alfa shows promise as a novel treatment for nAMD, potentially offering improved efficacy over current anti-VEGF therapies. Nonetheless, further large-scale randomized clinical trials are essential to confirm its efficacy and safety in broader populations. The dual-inhibition strategy provides a new avenue for personalized AMD treatment, particularly for patients unresponsive to monotherapies.

## Introduction

Age-related macular degeneration (AMD) is a condition that causes progressive vision loss, most commonly affecting individuals over 50 years of age. It is a leading cause of irreversible blindness in people over 65 years [1]. It is estimated that 200 million people globally are currently affected by AMD, with this number projected to rise to nearly 300 million by 2040. In the U.S. alone, 5.4 million Americans are expected to be impacted by 2050, following similar global trends. While people of Caucasian descent are the most affected, research suggests that intermediate dry AMD and wet choroidal vasculopathy are more prevalent among Asian and African American populations [2]. Because of its chronic nature and the need for ongoing long-term management, AMD has emerged as a persistent public health issue in both high- and low-income countries. This condition brings significant socioeconomic challenges and contributes to rising healthcare costs worldwide [2]. In the late twentieth century, neovascular AMD (nAMD) accounted for only 10% of the total cases but was responsible for 90% of AMD-related legal blindness [3].

AMD is caused by the deposition of drusen between Bruch’s membrane and neurosensory retina. Drusen are composed of lipids, proteins, and carbohydrates, resulting from the accumulation of uncleared cellular debris produced by the retinal pigment epithelium [4]. Drusen vary in size from 20 to 100 µm and may be either soft or hard. Hard drusen are round, well-defined yellow deposits that are neither age-related nor associated with elevated risk of neovascularisation. On the other hand, soft drusen are poorly defined, lack discrete borders, and are associated with a high risk for progression to neovascular AMD [5].

Visualization of drusen on dilated fundus examination is the primary sign of the dry form of AMD. They appear as yellow spots on examination. As drusen accumulate, they impair the transport of nutrients and waste materials to and from the choroid, leading to vascular compromise [4]. An increase in the number and size of drusen is associated with a higher risk of disease progression [6].The disease may progress to the wet form of AMD, characterised by neovascularisation within the macula [4]. This change is brought upon by the recruitment of immune cells to the site of macular damage, which initiates the release of pro-inflammatory and pro-angiogenic mediators, particularly vascular endothelial growth factor (VEGF), which in turn stimulates the formation of new blood vessels.

The diagnosis of AMD is typically made clinically, but can also be done with fundus autofluorescence, optical coherence tomography, fluorescein angiography, and indocyanine green angiography [5]. The Macular Research Classification Committee (MRCC) has recently proposed a consensus classification of AMD based on the presence of lesions within two disc diameters of the fovea in either eye, ranging from stage 1 to stage 5 [1].

Treatment strategies involve laser photocoagulation, photodynamic therapy, radiotherapy, and angio-static steroids. These approaches were largely limited to preventing disease progression and did not restore diminished visual function [7].

One of the most significant advances in ophthalmology in the past 50 years has been the discovery that VEGF mediates choroidal neovascularisation, thus providing a potential therapeutic target [8]. Approximately 1 million patients receive VEGF inhibitor treatments annually, including those with nAMD and other retinal diseases associated with abnormal blood vessel growth [8].

Before the introduction of anti-VEGF therapy, thermal laser coagulation was used to eliminate drusen deposits. However, it often caused damage to the surrounding tissues, resulting in further destruction and frequent recurrences. Recent advances have led to the development of short-pulse lasers, which are sufficient to destroy drusen without harming neighboring structures [9, 10].

Efdamrofusp alfa is an innovative bispecific decoy receptor fusion protein that independently neutralizes both C3b/C4b and VEGF. It has shown anti-angiogenic effects in models of laser-induced choroidal neovascularization (CNV) in mice and non-human primates. Additionally, it displayed a favorable safety profile and was well tolerated in a first-in-human trial (NCT03814291) [11, 12]. Due to its dual mechanism of action on angiogenesis and the complement system, this novel drug has the potential to be very effective in nAMD. However, conclusive evidence regarding the same is still lacking.

This review aims to explore the current status of the novel drug and explore the barriers to its full integration into clinical practice.

## Efdamrofusp Alfa: pharmacological profile

### Physiology

Vascular endothelial growth factor A (VEGF-A) and its receptors (VEGFR1 and VEGFR2) have been shown to contribute significantly to angiogenesis, whether it is a physiologic process (eg, during embryogenesis) or part of a disease pathology (eg, tumors) [13]. VEGF receptors are Tyrosine kinase receptors (TKRs) that include an extracellular domain for ligand binding, a transmembrane domain, and a cytoplasmic domain, including a tyrosine kinase domain [14].

The complement system has wide-ranging effects on the human body, [15], one of which has been recognized recently, its involvement in angiogenesis [16]. Active components of complement proteins, such as C3a and C5a, have been found in drusen in AMD patients, [17] which are one of the earliest hallmarks [18]. Other clinical studies have also shown the complement system to affect angiogenesis in various ways [19, 20].

### Mechanism of action

**Fig. 1:**
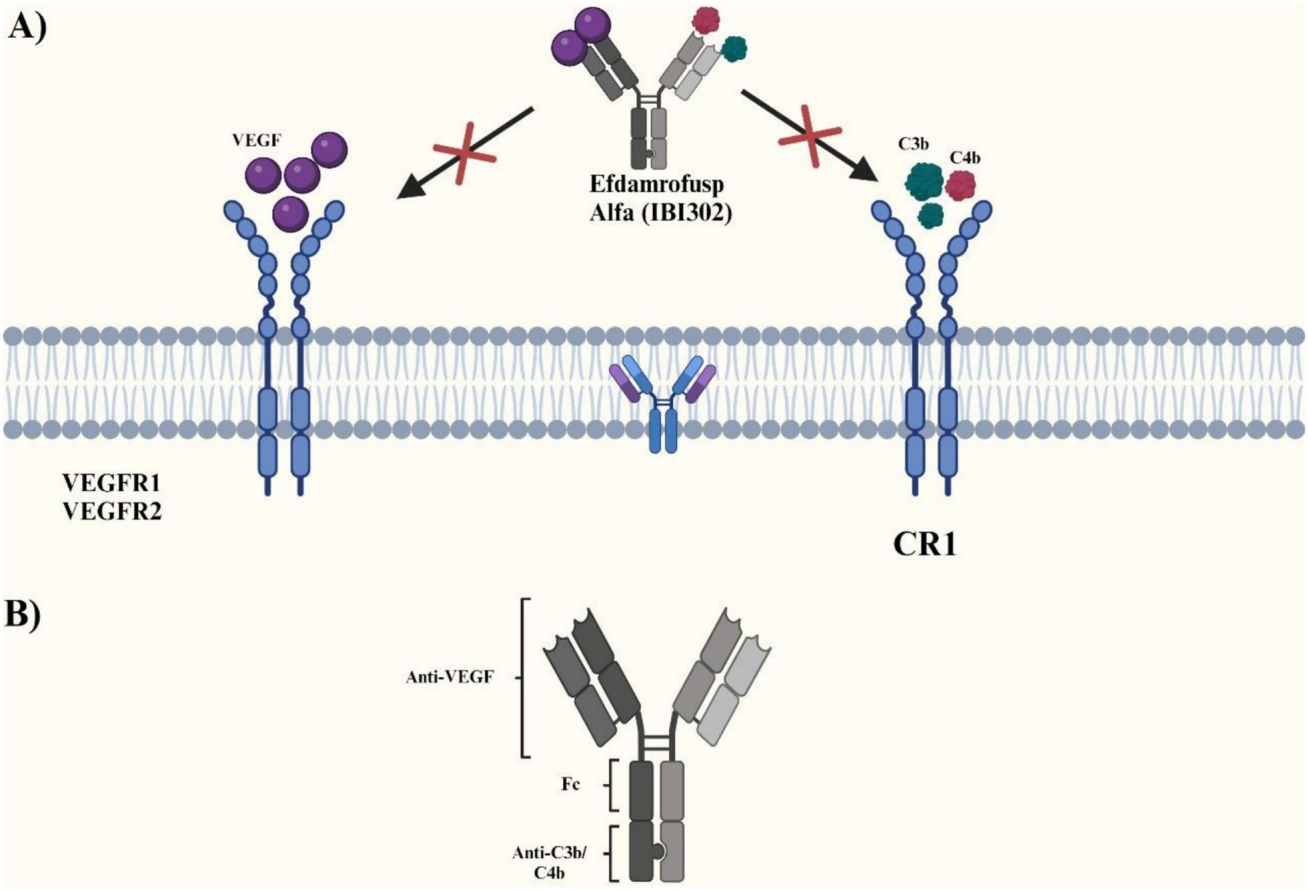
Mechanisms of action of Efdamrofusp alfa: A. Target across cell membrane, B. Structure of the bispecific protein

Efdamrofusp Alfa (IBI302) is a novel bispecific decoy receptor fusion protein that targets both VEGF and complement domains, which are connected by the Fc region of the human immunoglobulin (Fig. 1) [11]. The VEGF domain combines extracellular domain 2 of VEGFR1 and extracellular domain 3 of VEGFR2, while the complement cascade inhibition domain includes three C3b and C4b binding sites of complement receptor 1 (CR1) [11]. Moreover, its anti-angiogenic property has been attributed to decreased macrophage recruitment and reduced M2 macrophage polarization, in addition to local inhibition of complement [12].

### Pharmacokinetics and pharmacodynamics

The pharmacokinetic and pharmacodynamic properties of Efdamrofusp Alfa (IBI302) have been summarised in the table below (Table 1).

**Table 1:** Pharmacokinetics of Efdamrofusp alfa

Study	Dose and route of Administration	Maximum concentration (Cₘₐₓ)	Time at maximum concentration (Tₘₐₓ)	Half-life (t₁/₂)	Bioavailability
Ren et al. [11]	Group 1 Intravitreal (0.5 mg/eye)	5.9 ng/mL	6 h	41 h	30.90%
	Group 2 Intravenous (1 mg/animal)	238 ng/mL	0.6 h	9.4 h	NA
Yang et al. [12]	Intravitreal (0.05 mg to 4 mg)	NA	NA	NA	NA
Jia et al. [21]	Intravitreal (2 mg/eye)	14.7 pmol/L	4.08 h	NA	NA
Sun et al. [22]	Intravitreal (2 mg and 4 mg)	NA	NA	NA	NA
Liu et al. [23]	Intravitreal (2 mg)	Ongoing trial			

NA: no information available

## Clinical implications

As people age and live longer, AMD-one of the main causes of blindness- is becoming an increasingly significant public health concern. Agents that target vascular endothelial growth factor (VEGF) through different mechanisms, like bevacizumab (used off-label), aflibercept, faricimab, brolucizumab, and ranibizumab, have revolutionized the treatment of nAMD, becoming the standard of care and offering a range of therapeutic options [22].

Research has shown that, even after one year of treatment, 35.2% to 70.9% of individuals with nAMD continued to have intraretinal or subretinal pigment epithelium (RPE) fluid [24–26]. Furthermore, despite the strictest regimens of anti-VEGF monotherapy, follow-up studies revealed that, in two-thirds of patients, visual improvements achieved during the first two years were not sustained by the fifth or seventh year [27, 28]. This may be partially explained by the development of atrophy, increasing lesion fibrosis, and/or recurrent leakage [29–31]. So, developing new therapies to combat nAMD is essential. Multi-target drugs may offer more sustained efficacy because several pathways beyond VEGF signaling-such as the complement system, BMP9/ALK1 signaling, and erythropoietin signaling-have been linked to the etiology of nAMD [21].

**Fig. 2:**
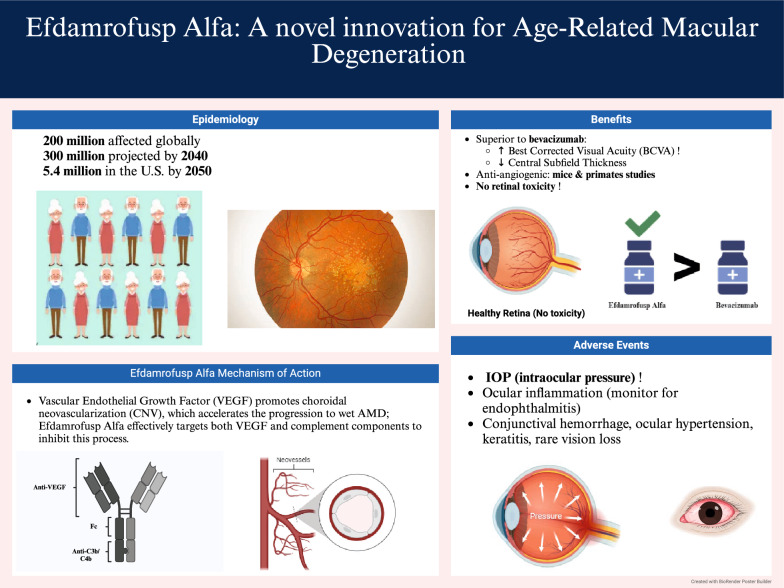
Summary of all findings on Efdamrofusp alfa in neovascular age-related macular degeneration

By simultaneously inhibiting VEGF and complement activation, the bispecific fusion protein efdamrofusp alfa has demonstrated improved efficacy over anti-VEGF monotherapy [12]. It has also shown an overall favorable safety profile in patients with nAMD (Fig. 2) [21]. In terms of best-corrected visual acuity (BCVA) improvement, efdamrofusp alfa showed noninferiority to aflibercept, with a comparable safety profile and also demonstrated significant reductions in central subfield thickness [12, 22].

A summary of all primary studies on efdamrofusp alfa is presented in Table 2. A Phase 1 multi-center, open-label, single-dose escalation clinical trial demonstrated that a single intravitreal injection of efdamrofusp alfa at doses ranging from 0.05 mg to 4.00 mg was well tolerated, with no significant ocular or systemic adverse effects. No dose-limiting toxicities (DLTs) were observed. The progressive reduction in the number of choroidal neovascularisation (CNV) lesions in eyes treated with efdamrofusp alfa was monitored using Optical Coherence Tomography (OCT) and Haematoxylin & Eosin (H&E) staining [12]. These findings emphasize the potential of Efdamrofusp alfa usage in routine practice for the treatment of nAMD.

**Table 2:** Summary of pre-clinical and clinical trials on the efficacy of Efdamrofusp Alfa for neovascular age-related macular degeneration (nAMD)

Reference	Status	Study design	Sample size	Dose of efdamrofusp alfa	Comparator	Key outcomes
Ren et al. [11]	Completed	Prospective randomized, open-label, multiple ascending-dose, phase 1b study	Total Monkeys: 27	Group 1 (IVT): 21 monkeys, 0.5 mg Group 2 (IV): 6 monkeys, 1 mg	Aflibercept Bevacizumab	High binding affinity to VEGF and complement proteins, favorable PK properties in ocular tissues, effective VEGF and complement pathway inhibition.The results indicate that IBI302 and aflibercept demonstrate higher inhibition potency compared to bevacizumab
Yang et al. [12] NCT03814291	Completed	Preclinical Study (In vitro, HUVEC migration assay & the tube formation assay)	Not applicable	0.135 mg/ml	Aflibercept (0.100 mg/ml) CID(0.092 mg/ml) IgG1(Control group)	Efdamrofusp alfa showed a 20.91% reduction in VEGF-induced migration of HUVECs, similar to Aflibercept (24.10% reduction). No significant difference between treatments. Treating HUVECs with CID did not significantly reduce their migration rather than IgG1 group VEGF administration stimulated endothelial cell tube formation when HUVECs were cultured both Efdamrofusp alfa & Aflibercept played an inhibitory role on the growth of endothelial tube formation. However, CID did not show any inhibitory effects on HUVECs tube formation. Efdamrofusp alfa reduced tube length in 33.80% and afliberecept reduced tube length in around 32.09%
		Preclinical Study (CDC assay, Raji cells)	Not applicable	EC50 of 5.322 µg/ml	Afliberecept CID (EC50 of 5.322 µg/ml)	Efdamrofusp alfa and CID significantly inhibited complement activation in a dose-dependent manner; Aflibercept did not show complement inhibition
		Preclinical Study (mouse model of laser-induced CNV)	16 CNV(4 mice per group)	13.5 μg	IgG1 (13.0 μg) Aflibercept (10.0 μg) CID (9.2 μg)	Efdamrofusp alfa significantly reduced CNV area, complement activation, and macrophage infiltration more effectively than Aflibercept. Three days after intravitreal treatment, efdamrofusp alfa or CID (a complement inhibitor) markedly decreased MAC (C5b-9 complex) deposition in the RPE-choroid complex, while Aflibercept did not. Additionally, efdamrofusp alfa significantly reduced the deposition of C3d, a marker of complement activation, compared to control, indicating its stronger effect in suppressing local complement activity in the laser-induced CNV model
		Preclinical Study (Nonhuman primates, CNV)	12 rhesus monkeys (4 groups)	1.35 mg	IgG1 (1.30 mg) Aflibercept (1.00 mg) CID (0.92 mg)	Efdamrofusp alfa, Aflibercept, and CID all effectively reduced CNV leakage and neuroretinal edema, with efdamrofusp alfa showing a greater reduction in complement deposition and inflammation. Fourteen and 28 days after injection, both efdamrofusp alfa and Aflibercept significantly decreased CNV leakage, with severity scores reduced by 1.352 and 1.130, respectively, compared to the control. Additionally, all three treatments reduced CNV volume at 28 days post-treatment
		Transcriptomic analysis of efdamrofusp alfa–mediated antiangiogenic effects in mouse model of CNV	N/A	13.5 μg	Aflibercept (10.0 μg)	The study comparing efdamrofusp alfa and aflibercept in a laser-induced CNV model showed that efdamrofusp alfa had a broader impact on immune modulation. Efdamrofusp alfa altered the expression of 1121 genes, significantly more than aflibercept, with many of these genes involved in immune-related processes. Key pathways affected by efdamrofusp alfa included macrophage activity, IL-6 signaling, and leukocyte extravasation, which were less influenced by aflibercept. Efdamrofusp alfa also centered around the regulation of arginase-1 (ARG-1), a gene linked to macrophage polarization, suggesting stronger immune modulation and anti-inflammatory effects compared to aflibercept
		Retinal Toxicity Study post intravitreal injectons in Mice	11(6 mice for scotopic ERG &5 mice for photopic ERG analysis)	13.5 μg	IgG1 (13.0 μg) Aflibercept (10.0 μg) CID (9.2 μg)	The study found that intravitreal administration of efdamrofusp alfa did not cause retinal toxicity. Histological analysis showed no changes in retinal morphology 7 days post-injection, and electroretinography (ERG) revealed no reduction in retinal function (a-wave and b-wave amplitudes) 28 days post-injection. These results indicate that efdamrofusp alfa is safe for the retina, with no signs of toxicity
		Phase 1 dose-escalation study	31 patients with nAMD (6 cohorts)	0.05 mg – 4.00 mg	None	Demographics: Age: 66.4 years (mean); BCVA: 34–64 letters; CST: 461.0–559.0 µm; CNV size: 1.460–7.200 mm [2]. Safety: No dose-limiting toxicity; TEAEs mild (74.2%); most common: conjunctival hemorrhage (48.4%). Outcomes: BCVA improved by + 6.0 letters (day 29); CST reduced by − 141.1 µm (day 29).Higher doses (0.50 mg or more) led to greater reductions in CST, with the 4.00-mg group showing the most significant anatomical improvements
Jia et al. [21]	Completed	Phase 1b Clinical Study of Efdamrofusp Alfa in nAMD	18 patients (3 cohorts, 6 patients each)	Efdamrofusp alfa 2 mg and 4 mg at weeks 0, 4, 8	Aflibercept 2 mg at weeks 0, 4, 8, and 16	BCVA Gain at Week 20: + 5.64 letters (2 mg), + 8.93 letters (4 mg), + 7.92 letters (Aflibercept). CST Change at Week 20: − 157.53 μm (2 mg), − 148.61 μm (4 mg), − 176.69 μm (Aflibercept). CNV Area Reduction: − 1.02 mm^2^ (2 mg), − 2.39 mm^2^ (4 mg), − 1.39 mm^2^ (Aflibercept)
Sun et al. [22]	Completed	Phase 2 Randomized, Double-Masked, Non-Inferiority Trial	231 participants with active CNV secondary to nAMD	2 mg Efdamrofusp alfa, 4 mg Efdamrofusp alfa	2 mg Aflibercept	BCVA Change (Week 36): + 10.6 letters (2 mg), + 11.4 letters (4 mg), + 12.0 letters (Aflibercept); Noninferiority Margins: LS mean difference of –1.4 (80% CI: –3.5 to 0.7) between 2 mg Efdamrofusp alfa and Aflibercept. Adverse events comparable across all groups
Liu et al. [23]	Ongoing	A Phase 3, Randomized, Double-Masked, Active-Controlled Study to Evaluate the Efficacy and Safety of Intravitreal IBI302 (Efdamrofusp Alfa) in Subjects With Neovascular Age-Related Macular Degeneration	600	8 mg	Aflibercept (2 mg/eye, Q4W for 3 months, then Q8W)	Change in BCVA from baseline at Weeks 44, 48, 52 Absence of intraretinal and subretinal fluid at Week 16, changes in BCVA, CST, CNV area, and treatment intervals up to Week 100 Incidence and severity of ocular and non-ocular adverse events, treatment-emergent adverse events (TEAEs) up to Week 100

*Abbreviations*: IBI302:Efdamrofusp Alfa VEGF: Vascular Endothelial Growth Factor CID: Complement Cascade Inhibition Domain IgG1: Immunoglobulin G subclass 1 IVT: Intra vitreal Injection IV: Intravenous PK: Pharmacokinetics nAMD: Neovascular Age-Related Macular Degeneration BCVA: Best Corrected Visual Acuity CST: Central Subfield Thickness CNV: Choroidal Neovascularization TEAE: Treatment-Emergent Adverse Event IOP: Intraocular Pressure SRF: Sub retinal Fluid HUVEC: Human Umbilical Vein Endothelial Cells

While the safety profile aligns with existing anti-VEGF therapies, [32] targeted strategies are required to mitigate treatment-related toxicities. The adverse events reported to be associated with Efdamrofusp alfa can be categorised into (12,21) treatment-emergent adverse events and drug-limiting toxicities (Table 3):

**Table 3:** Adverse events noted on use of Efdamrofusp alfa

Treatment-emergent adverse events (TEAE)	Dose-limiting toxicities
1. Conjunctival hemorrhage 2. Keratitis 3. Conjunctival hyperemia 4. Retinal hemorrhage 5. Ocular hypertension (Associated with Intra-vitreal drug administration)	1. Intra-ocular inflammation 2. Sustained elevation of intra-ocular pressure (> = 10 mmHg) for at least 60 min 3. Acute vision loss uncorrelated with intraocular inflammation 4. Moderate or severe vitreous hemorrhage 5. Intracranial hemorrhage or other extra-ocular haemorrhage of clinical significance

### Intraocular pressure elevation/Ocular hypertension

Intraocular injection-related acute rise in intraocular pressure (IOP) following intravitreal injection typically lasts a few hours [33, 34]. However, following the first and second doses of efdamrofusp alfa at 4 mg, one patient experienced a brief, asymptomatic increase in intraocular pressure [21]. It is recommended that all patients undergoing intravitreal therapy undergo regular IOP monitoring, with topical or surgical anti-glaucoma interventions as needed [35–37].

### Intraocular inflammation

Distinguishing infectious inflammation (including endophthalmitis) from non-infectious, sterile ocular inflammation can be challenging [32]. Any case of uveitis following intravitreal injection should be treated as suspected endophthalmitis, as clinical symptoms may overlap or even appear more severe than those of acute sterile inflammation. When there is a high degree of clinical suspicion, intravitreal antibiotics should be administered [38, 39].

These findings collectively suggest that efdamrofusp alfa holds promise as a next-generation treatment for nAMD, particularly in patients who show suboptimal response or durability with traditional anti-VEGF monotherapies. While early-phase studies indicate favorable safety and efficacy profiles, further large-scale, long-term trials are essential to establish its real-world effectiveness and safety across diverse patient populations. In clinical practice, its dual mechanism of action could lead to reduced injection frequency, improved visual outcomes, and potentially lower the burden on both patients and healthcare systems.

As the treatment for nAMD shifts toward multi-targeted approaches, it is important to compare efdamrofusp alfa with other emerging agents beyond standard anti-VEGF therapies. In addition to faricimab—which targets both VEGF-A and Angiopoietin-2 [40]—newer agents such as KSI-301 (tarcocimab tedromer), a novel anti-VEGF antibody biopolymer conjugate [41], and RGX-314, a gene therapy delivering anti-VEGF proteins via subretinal or suprachoroidal administration [42], offer alternative strategies aimed at reducing treatment frequency and improving long-term outcomes. Furthermore, OPT-302, a VEGF-C/D trap, shares mechanistic overlap with efdamrofusp alfa in targeting VEGF-C, raising questions about synergistic or competitive effects [43]. Comparing efdamrofusp alfa’s efficacy, durability, safety profile, and mechanism of action against these novel therapies will be essential to position it within the future therapeutic landscape of AMD. Head-to-head clinical trials, real-world studies, and network meta-analyses will be critical to elucidate its relative advantages.

## Challenges and limitations

Efdamrofusp alfa holds the potential to revolutionize the management of nAMD. However, like any emerging therapeutic agent, its path to widespread clinical adoption requires validation through well-designed and rigorously executed clinical trials. To date, research on efdamrofusp alfa remains limited, with only a handful of clinical studies available. Most of these, including those by Yang et al., Jia et al., and Sun et al. [12, 21, 44], feature relatively small sample sizes, limiting their statistical power and generalizability. Therefore, larger, multicenter randomized clinical trials and longitudinal cohort studies are urgently needed to establish the safety, efficacy, and long-term benefits of efdamrofusp alfa in the treatment of nAMD.

Early trials have reported several treatment-emergent adverse events (TEAEs), including conjunctival hemorrhage, ocular hypertension, keratitis, and ocular hyperemia [12, 21]. Notably, a case of six-letter visual acuity loss was observed in a patient who received a 2 mg injection of efdamrofusp alfa [12]. Although these adverse events appear to be rare, their incidence may become more prominent in larger and more diverse populations, potentially affecting patient compliance and enrollment in future trials. This underscores the importance of thoroughly evaluating the risk–benefit profile of efdamrofusp alfa.

Additionally, the current trials have not adequately addressed key biological variables such as genetic polymorphisms. Given that nAMD is influenced by a wide range of genetic factors that can significantly affect individual responses to treatment [12], the lack of stratification or analysis based on genetic variation limits the applicability of current findings and hinders the development of personalized therapeutic strategies.

Several other limitations and challenges must also be considered. First, the follow-up periods in existing studies are generally short, preventing a clear understanding of the long-term efficacy and safety of efdamrofusp alfa. Second, the absence of direct comparisons with current standard-of-care treatments—such as aflibercept or ranibizumab—hampers efforts to position efdamrofusp alfa within the current therapeutic landscape. Third, issues related to cost-effectiveness, accessibility, and patient-reported outcomes remain largely unexplored, all of which are critical for real-world adoption.

## Future directions

In the latter part of the previous century, treatments such as macular translocation surgery, laser photocoagulation, and photodynamic therapy emerged for the management of nAMD. However, these were eventually superseded by anti–VEGF agents, which offered a less invasive and more patient-friendly approach [45]. This paradigm shift was driven by a combination of robust research, innovation, and clinical need. Today, a new generation of innovative therapies, including efdamrofusp alfa, is being explored for the treatment of nAMD.

Efdamrofusp alfa represents a promising therapeutic advance [12], but several shortcomings must be addressed. Future research should prioritize minimizing TEAEs such as conjunctival hemorrhage, keratitis, conjunctival hyperemia, retinal hemorrhage, and ocular hypertension [21, 22]. It is important to note that many of these TEAEs are likely associated with the intravitreal route of administration rather than the drug itself [32], highlighting the need for the development of less invasive drug delivery methods.

Moreover, rigorous scrutiny of study designs and reporting is essential to ensure comprehensive documentation of less frequently observed TEAEs, such as increased intraocular pressure and intraocular inflammation. These may act as dose-limiting toxicities, and meticulous monitoring is essential for advancing the therapeutic potential of efdamrofusp alfa [12, 21]. In parallel, effective patient counseling regarding possible adverse events, the importance of regular follow-up, and the anticipated long-term benefits of therapy will be critical for improving adherence and overall treatment safety.

Furthermore, the unique mechanism of action of efdamrofusp alfa—targeting both inflammation and neovascularization—suggests its potential application in other retinal conditions such as geographic atrophy, diabetic retinopathy, retinal vein occlusion, and retinal fibrosis [44]. Future studies should explore these possibilities, particularly in conjunction with novel administration routes, which may reveal additional therapeutic targets. A broader research agenda may also examine its applicability beyond ophthalmology, potentially extending its utility to other organ systems.

Finally, while efdamrofusp alfa offers substantial promise, it is important to recognize that other therapeutic modalities, such as gene therapy, stem cell therapy, and protein-based treatments, are also under investigation. Although some of these are in early phases of development, further extensive research is necessary to accelerate their clinical translation [31].

## Conclusion

Efdamrofusp alfa represents a significant advancement in the treatment of neovascular age-related macular degeneration. The dual inhibition of VEGF and complement pathways offers a promising alternative to existing mono-targeted therapies, potentially improving both efficacy and safety profiles. While early-stage studies demonstrate encouraging results, further large-scale clinical trials are essential to establish its long-term benefits and confirm its potential to transform AMD management. By addressing the multifaceted nature of AMD, Efdamrofusp alfa may pave the way for more effective, personalized treatments, ultimately reducing the burden of this debilitating condition on patients and healthcare systems worldwide.

## Data Availability

No datasets were generated or analysed during the current study.
